# Deep learning classification of nasal anatomical variants on computed tomography for endoscopic sinus surgery

**DOI:** 10.1038/s41598-026-59617-1

**Published:** 2026-07-01

**Authors:** Luís Rita, Abdul Rafay, Hamideh Kerdegari, Matth Kot, Ivan Laponogov, Kyle Higgins, Dennis Veselkov, Issa Beegun, Hesham Saleh, Kirill Veselkov

**Affiliations:** 1 Division of Cancer, Department of Surgery and Cancer, Faculty of Medicine, Imperial College London, London, W12 0NN UK; 2 Department of Rhinology, Faculty of Medicine, Imperial College London, London, SW7 2AZ UK; 3 Department of Computing, Faculty of Engineering, Imperial College London, London, SW7 2AZ UK; 4 Department of Environmental Health Sciences, Yale University, New Haven, CT 06520 USA

**Keywords:** Paranasal sinuses, Computed tomography, Deep learning, Convolutional neural networks, Anatomic variation, Vision-language models, Computational biology and bioinformatics, Diseases, Medical research

## Abstract

Endoscopic sinus surgery (ESS) carries risks such as vision loss and intracranial injury due to the proximity of critical structures and unrecognised anatomical variants. We developed convolutional neural networks (CNNs) to detect free-floating anterior ethmoidal arteries (FFAEA), Onodi cells, and Haller cells on coronal sinus CT, and evaluated BioMedCLIP, a biomedical vision-language model (VLM), in a few-shot setting. CT scans from 122 ESS patients were anonymised, standardised coronal CT images were captured, and variant presence was annotated. Five ImageNet-pretrained CNN backbones were assessed using repeated patient-wise five-fold cross-validation across 40 configurations. The best CNNs achieved balanced accuracies of 77.8 ± 2.3% (FFAEA), 74.6 ± 2.5% (Onodi), and 63.7 ± 6.2% (Haller). BioMedCLIP achieved 65.5 ± 3.2%, 63.8 ± 1.8%, and 73.5 ± 3.6%, respectively, outperforming CNNs for Haller cell detection while providing competitive performance for the other variants. These models demonstrate proof-of-concept performance for automated identification of selected sinonasal variants on standardised coronal CT images under internal patient-wise cross-validation.

**Supplementary Information:**

The online version contains supplementary material available at https://doi.org/10.1038/s41598-026-59617-1.

## Introduction

Endoscopic sinus surgery (ESS) - often referred to as functional endoscopic sinus surgery (FESS) - is a minimally invasive endoscopic procedure performed to access and treat diseased paranasal sinuses. It is primarily indicated for chronic rhinosinusitis (CRS) that does not respond to medical therapy but is also employed in the management of several sinonasal and skull base pathologies, with the aim of restoring normal sinus drainage and ventilation^1^. Approximately 19,000 ESS procedures are performed each year within England’s National Health Service (NHS), making it one of the most common operations in otolaryngology^2^. National data shows a 21% increase in ESS procedures between 2010 and 2019, reflecting a growing surgical burden^3^.

Though it is an established procedure, major complications - such as cerebrospinal fluid (CSF) leak, orbital injury, and massive haemorrhage - can occur in up to 1% of ESS cases^4^. This risk is largely attributed to the close proximity of vital structures to the operative field, including the skull base, orbit, and internal carotid arteries^5^. Consequently, pre-operative computerised tomography (CT) imaging of the paranasal sinuses is routinely performed to map this sensitive area^6^. The anatomy of this region can vary considerably between individuals, with differences in the size, shape, and position of key structures. While these naturally occurring differences - known as anatomical variants - are not pathological, they can have important clinical and surgical implications^7^. Certain nasal anatomical variants are known to increase the likelihood of inadvertent injury during ESS. These include, but are not limited to, a free-floating anterior ethmoidal artery (FFAEA), Haller cells and Onodi cells.

The anterior ethmoidal artery (AEA), a branch of the ophthalmic artery, traverses the ethmoid sinus before entering the anterior cranial fossa^8^. On CT, its location is typically inferred by identifying the anterior ethmoidal notch in the coronal plane, visible in 95–100% of CT sinus scans^9^. Notably, the AEA exhibits considerable anatomical variation, particularly in its vertical position relative to the ethmoid roof - ranging from closely adherent to the skull base to freely hanging within the ethmoid sinus^8,10,11^. This *free-floating* variant appears in 20–42% of CT sinus scans and, if unrecognised preoperatively, increases the risk of iatrogenic injury to the artery during ESS^11–13^.

The Haller cell is an infraorbital ethmoid air cell located along the orbital floor. It is identified on CT in approximately 10–30% of patients and, due to its position, places the orbit at risk of inadvertent entry during ESS^14,15^. Additionally, this variant may obstruct access to the maxillary sinus or anterior ethmoidal cells during the procedure, limiting exposure to the operative field^16,17^. The Onodi cell, or sphenoethmoidal air cell, is a posterior ethmoidal cell that extends superiorly or posteriorly into the sphenoid sinus. It is identified in approximately 8–33% of CT sinus scans^14,18^. During ESS, failure to recognise an Onodi cell may result in inadvertent penetration of its posterior wall, placing adjacent critical structures - namely the optic nerve, pituitary fossa, and internal carotid artery - at risk of serious injury^14,19^.

Given the potential for anatomical variation, pre-operative CT evaluation of the paranasal sinuses is essential for planning ESS and minimising the risk of complications^11,20,21^. However, there are currently no published radiological or otolaryngological guidelines that define a standardised approach to the interpretation of CT sinus scans for pre-operative planning. In addition, radiological reporting has often been found to lack sufficient anatomical detail. A multi-institutional review found that 22% of otolaryngologists were dissatisfied with current sinus CT reports, and 67% expressed a desire for more clinically relevant information. Notably, the location of the AEA and the presence of Onodi and Haller cells were mentioned in only 2%, 7%, and 10% of reports, respectively^22^. This makes the prospect of an artificial intelligence (AI) algorithm that aids radiological reporting by flagging clinically relevant nasal anatomical variants an attractive possibility.

AI refers to the broader field of computer science dedicated to developing systems capable of performing tasks that typically require human intelligence, such as decision-making and pattern recognition. Machine learning (ML), a subset of AI, involves algorithms that learn from data to improve their performance in making predictions or decisions without being explicitly programmed. Deep learning (DL) is a further subset of ML that utilises artificial neural networks - computational models inspired by the structure of the human brain - to automatically learn complex patterns within large datasets. Convolutional neural networks (CNNs) are a specific type of DL architecture particularly well-suited to analysing visual data, and have become increasingly popular in the field of radiology^23^.

Previous studies have successfully applied CNNs in head and neck radiology, primarily for tumour detection and prognostication across various imaging modalities, including PET, CT, and MRI^23–25^. In the field of rhinology, CNNs have been developed to detect and segment both normal structures and anatomical variants on CT sinus scans^26–28^. However, no studies have yet developed models targeting anatomical variants with direct clinical relevance to ESS.

Recently, a new class of models known as vision-language models (VLMs) has emerged, which jointly process visual and textual data, enabling generalisable reasoning across modalities. These models offer a paradigm shift from conventional supervised learning by leveraging paired image-text data to acquire semantically rich, transferable representations. Their ability to integrate visual and linguistic cues allows them to perform a wide array of tasks without task-specific retraining, making them particularly appealing in data-scarce clinical scenarios^29^. Among them, BioMedCLIP stands out as a biomedical-domain-adapted VLM that capitalises on large-scale medical corpora and radiological image-text pairs. Its architecture supports zero-shot and few-shot inference, facilitating rapid deployment in diagnostic workflows where annotated data are limited or unavailable. Integrating such models into radiology pipelines could enhance the detection of anatomically complex or rare variants that are frequently underreported in conventional settings. BioMedCLIP, a large-scale VLM trained on biomedical literature and imaging pairs, is among the most promising of these models for clinical tasks with limited labelled data^30^.

As such, the primary aim of this study is to explore the use of artificial intelligence methods, including both CNNs and VLMs, to identify anatomical landmarks relevant to ESS on CT sinus scans. Specifically, we investigate the detection of three clinically significant anatomical variants: FFAEA, Onodi cells, and Haller cells. To our knowledge, this is the first study to comprehensively evaluate both CNN-based architectures and foundation VLMs in this domain. The contributions of this study are threefold: (1) we develop and benchmark multiple CNN architectures using patient-wise cross-validation for robust variant detection; (2) we introduce the application of BioMedCLIP, a biomedical-domain VLM, for few-shot classification of sinonasal anatomical structures; and (3) we provide an open-source framework and annotated dataset to facilitate reproducibility and future development in AI-assisted ESS planning. This work establishes a proof-of-concept framework for future DICOM-native, multi-planar, and externally validated AI tools that may support pre-operative radiological workflows in otolaryngology.

## Methods

This study followed the Checklist for Artificial Intelligence in Medical Imaging (CLAIM) guidelines, established by the Radiological Society of North America, to ensure standardised, transparent, and reproducible reporting of the study’s methodology and results^31^. The completed checklist can be found in the Appendix A. These guidelines were applied to both the supervised CNN training pipeline and the inference-only VLM workflow.

### Data acquisition and labelling

Patients referred for ESS at our centre were identified within secure hospital systems. CT sinus scans were retrieved from the hospital Picture Archiving and Communication System (PACS) between February and April 2025. Each case was assigned a randomly generated five-digit study code (*patientID*). Following retrieval, all scans and associated labels were fully anonymised prior to analysis, and the analytical dataset contained no direct or indirect patient identifiers.

For this proof-of-concept study, CT scans were reviewed within the hospital PACS viewer and de-identified 2D image captures were generated rather than exporting native DICOM files. This approach was selected because native DICOM files contain extensive embedded metadata and may include institution-specific or patient-related identifiers, requiring a substantially longer de-identification, verification, and information-governance process before use in a research dataset. The screenshot-based workflow enabled rapid generation of effectively anonymised image data while preserving the coronal anatomical appearances required for the classification tasks.

CT images were reviewed directly within the hospital PACS viewer using this de-identified image-capture workflow. Raw DICOM metadata were not extracted for the analytical dataset; therefore, the coronal reformat matrix, pixel spacing, slice thickness, and slice spacing were not recorded for individual scans. No spatial resampling was performed before image capture. Images were acquired at the native PACS viewing resolution using the default bone or sinus window settings available in PACS at the time of review; a fixed window width/level preset was not enforced.

De-identified coronal image captures were obtained from each CT scan, targeting three anatomical variants: FFAEA, Haller cells, and Onodi cells. These variants were selected because of their clinical relevance to ESS and because they can be visualised on coronal CT; however, in routine ESS planning, confirmation of Onodi cells and the exact course of the anterior ethmoidal artery often also relies on axial and sagittal reformats. The patientID was used to label all extracted images, which were saved in the format patientID.1, patientID.2, and so on. This allowed all images from a single patient to be grouped together during model training and validation, ensuring the model was never trained and validated on images from the same patient. It also served to maintain patient confidentiality when collaborating with non-medical personnel. No patient-identifiable data were recorded or visible on the extracted CT images.

Scans with mucosal thickening and partial or complete opacification of the ethmoid, sphenoid, and/or maxillary sinuses were included, reflecting the clinical population referred for ESS, many of whom had chronic rhinosinusitis. No scans were formally excluded solely based on sinus opacification or anatomical obscuration, although inflammatory change may have reduced the visibility of some target structures, particularly Haller cells.

Two sets of coronal CT images were captured per scan: an anterior and a posterior stack, each targeting distinct anatomical regions. For both stacks, the image boundaries, anatomical region of interest, and location of the most anterior image were standardised using predefined anatomical landmarks. A fixed slice interval between consecutive images was not enforced. Instead, additional posterior images were captured at locations judged by clinical expertise to best represent the relevant anatomy and surrounding spatial context. This multi-image strategy was used to preserve limited three-dimensional anatomical information in the absence of direct volumetric DICOM input and extends previous CT-sinus CNN approaches that commonly used a single landmark-based image per scan^26,32^.

The anterior stack, used to assess for FFAEA and Haller cells, was captured in the region of the osteomeatal complex (OMC). Screenshot boundaries were defined superiorly by the skull base or superior orbital border, laterally by the maxillary sinus walls, and inferiorly by the floor of the maxillary sinuses. The most anterior slice was selected as the first coronal section immediately posterior to the nasolacrimal duct, through the natural maxillary ostium. This location reflected approaches used in previous studies that also developed CNNs to detect anatomy in the region of the OMC^26,32^.

The posterior stack, used to assess for Onodi cells, was captured at the level of the sphenoid sinus. Screenshot boundaries were set superiorly at the anterior clinoid process, laterally by the sphenoid sinus walls, and inferiorly at the sinus floor. The most anterior slice in this stack was defined as the first image in which the anterior clinoid process could clearly be visualised.

**Fig. 1:**
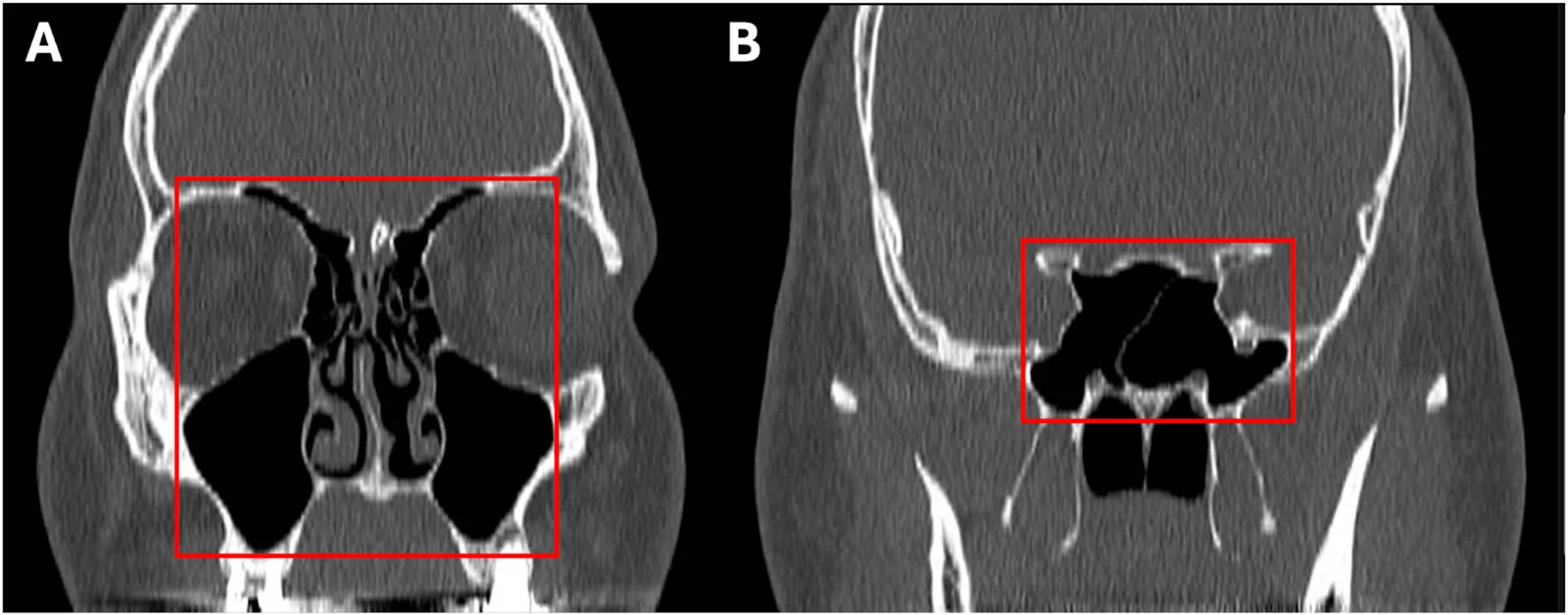
Coronal CT sinus scan from an anonymised patient demonstrating the most anterior slices used to define the anterior (**A**) and posterior (**B**) stacks. Additional coronal images were captured posterior to these slices where they best represented the normal or variant anatomy. Red boxes indicate the defined screenshot boundaries for each stack.

The screenshot boundaries on the most anterior slices for each stack are shown in Fig. 1.

The screenshot acquisition protocol was developed by the first author and validated by a consultant rhinologist, a professor in rhinology, and a PhD colleague specialising in AI.

For FFAEA annotation, no fixed millimetric threshold from the skull base was applied. Although previous studies have described the course of the anterior ethmoidal artery in relation to structures such as the skull base, ethmoidal bulla, and frontal beak^8,10,11^, there is no universally accepted radiological threshold defining a *free-floating* anterior ethmoidal artery. We therefore used expert visual consensus: the artery was classified as free-floating when the anterior ethmoidal artery/canal was observed to course through the ethmoid sinus rather than remaining adherent to the skull base or ethmoid roof. Initial categorisation was reviewed by a consultant rhinologist and discrepancies were resolved by independent review from a professor in rhinology.

Following acquisition, screenshots were categorised according to the presence or absence of anatomical variants. Three main folders were created - *FFAEA*, *Onodi cells*, and *Haller cells* - each subdivided into *Present* and *Not Present* classes. Initial screenshot collection and categorisation was performed by the first author. A consultant rhinologist, blinded to the initial classifications, then reviewed and re-categorised the scans. Any discrepancies were resolved through independent review by a blinded professor in rhinology.

### Model development (CNNs and VLMs)

CNNs and, separately, a pretrained VLM (BioMedCLIP) were evaluated; subsections below describe preprocessing, sampling, and model-specific workflows.

#### Image preprocessing and augmentation

All extracted CT images were converted to single-channel grayscale format and resized to 224 × 224 pixels to ensure a consistent model-input size. This resizing was applied to the extracted 2D image files and was distinct from DICOM voxel-level resampling; no spatial resampling was performed before image capture. Pixel values were then scaled and normalised to reduce variation in CT images brightness and contrast. Because the analytical inputs were derived from PACS-based 2D image capture rather than native DICOM volumes, preprocessing could not recover original Hounsfield unit information, acquisition metadata, or standardised window/level settings.

To enhance the model’s generalisability, the scan data was augmented using random horizontal flips, small rotations (up to ± 15°), and translations of up to 10% of the image dimensions to simulate natural variations in scan positioning.

For BioMedCLIP, images were instead converted to RGB and preprocessed using the model’s standard transforms, as required by the pretrained vision encoder.

#### Dataset and sampling

A custom dataset class was developed in PyTorch to load each CT image, apply the necessary image transformations, and assign the correct label. Labels were originally marked as *Present* or *Not Present* and then converted to binary format (1 for *Present*, 0 for *Not Present*) to match the input requirements of the CNN and to maintain consistency across models.

Class imbalance was present in the training data, with *Not Present* images outnumbering *Present* ones. To address this, a weighted random sampling method was used, assigning each class a sampling weight inversely proportional to its frequency. During training, the model processed the data in batches of 32 images, with all images randomly selected using the weighted sampler to ensure a more balanced representation of both classes. A batch size of 32 was chosen as it offers a practical balance between training speed, memory usage, and learning stability on standard GPUs.

#### CNN architectures and training configurations

We trained CNNs to distinguish between *Present* and *Not Present* classes by minimizing the binary cross-entropy loss, which quantifies the divergence between predicted probabilities and ground-truth labels. During training, network weights - parameters that govern how feature maps are processed - are iteratively updated via gradient descent: gradients indicate both the direction and magnitude by which each weight must change to reduce the loss. We evaluated five established CNN backbones - MobileNetV2, ResNet18, DenseNet121, VGG16, and SqueezeNet1.0 - each initialized with ImageNet-pretrained weights then adapted to accept single-channel (grayscale) inputs and output a binary classification. To optimize performance, we conducted a full factorial search over three key hyperparameters: the optimizer (Adam vs. stochastic gradient descent with momentum), learning rate (LR) (0.001 vs. 0.0005), and total epochs (15 vs. 25). Adam leverages both the current gradient and its history to adaptively scale updates, whereas SGD with momentum applies more uniform updates smoothed by past gradients. LR governs the magnitude of each weight update - higher rates accelerate convergence but risk overshooting, whereas lower rates promote stability at the expense of slower learning - while the number of epochs determines how many times the model traverses the entire training set, balancing between sufficient pattern acquisition and the danger of overfitting. Combining five architectures with two choices for each of the three hyperparameters yielded 5 × 2 × 2 × 2 = 40 distinct training configurations per anatomical variant.

#### Patient-wise repeated stratified five-fold cross-validation

We assessed model performance using repeated stratified five-fold cross-validation at the patient level. Patients, rather than individual CT images, were assigned to folds so that all images from a given patient were contained entirely within a single fold. Stratification was performed separately for each anatomical variant to preserve approximately the same patient-level *Present*/*Not Present* distribution across folds. In each iteration, four folds (approximately 80% of patients) were used for training and the remaining fold (approximately 20%) for validation. This ensured that the model was never trained and validated on CT images from the same patient. Appendix B illustrates this scheme.

To reduce bias from any single grouping of the data, the five-fold cross-validation procedure was repeated three times using different random seed values: 2025, 42, and 1234. Seeds are fixed numerical values used to initialise the random number generator in the CNN’s code, which controls stochastic operations such as patient shuffling. Although the seed values were randomly selected, they were kept constant and documented to ensure reproducibility - allowing others to replicate the exact data splits used during cross-validation. In total, three cross-validation cycles were performed for each of the 40 training configurations. Performance metrics for each configuration were reported as the mean and standard deviation across these three cycles.

The same patient-wise cross-validation splits were applied when evaluating BioMedCLIP to ensure fully aligned comparisons between model families.

#### VLM (BioMedCLIP) few-shot classification

To complement the CNN experiments, we evaluated BioMedCLIP (PubMedBERT-256, ViT-Base-Patch16-224) in a few-shot setting. The model was used without fine-tuning, and only the pretrained image encoder was applied. All coronal CT images were converted to RGB and preprocessed using the model’s standard transforms before being passed through the image encoder to obtain 512-dimensional feature embeddings.

For each anatomical variant, the BioMedCLIP classifier was evaluated using the same patient-wise repeated five-fold cross-validation splits used for the CNNs. Within each training fold, a small support set of *Present* and *Not Present* images was sampled from the training patients. Class prototypes were then computed by averaging the image embeddings within each class.

During evaluation, embeddings from the query images (held-out patients from the same fold) were compared to the two class prototypes using cosine similarity, and each image was assigned to the class with the highest similarity. No text prompts or language-encoder features were used in this workflow.

The resulting predictions were aggregated across all folds and repeats, and the same evaluation metrics (balanced accuracy, F1 score and AUC) were computed as for the CNNs.

### Model evaluation

#### Evaluation metrics

The performance of each CNN and BioMedCLIP was quantified using three widely accepted metrics: balanced accuracy, F1 score, and the area under the receiver operating characteristic curve (AUC). Balanced accuracy, defined as the arithmetic mean of sensitivity and specificity, equally weights the model’s ability to correctly identify both positive (*Present*) and negative (*Not Present*) cases, thus mitigating bias in imbalanced datasets. The F1 score, the harmonic mean of precision (the proportion of positive predictions that are correct) and recall (sensitivity, the proportion of actual positives correctly identified), offers a single measure that jointly considers false positives and false negatives. Finally, AUC evaluates overall discrimination performance by measuring the area under the ROC curve, which plots sensitivity against the false positive rate; an AUC of 1.0 indicates perfect separability, whereas 0.5 indicates no better than random classification.

#### Training and data visualization software

CNN models were trained using the PyTorch deep learning framework (version 2.6.0 with CUDA 12.4 support), running on high-performance NVIDIA GPUs when available or on standard CPUs when necessary. TorchVision (version 0.21.0) was used for image preprocessing, while NumPy (version 2.0.2) and Pandas (version 2.2.2) supported numerical and tabular data handling. BioMedCLIP was also implemented in PyTorch using the publicly released pretrained weights and was used for inference only, without further fine-tuning. Performance metrics were calculated using scikit-learn (version 1.6.1). Plots and figures were generated using Matplotlib (version 3.10.0), Microsoft Excel, BioRender and GraphPad Prism (version 10.4.2), with colourblind-friendly palettes applied where applicable. Basic image file handling was performed using the Python Imaging Library (PIL, version 11.1.0).

## Results

CT scan data were collected from 122 patients, yielding 915 standardised images in total: 411 anterior-stack images and 504 posterior-stack images. Appendix C provides a detailed breakdown of patient numbers and image distribution across anatomical variants. Each of the 40 CNN training configurations was evaluated on all three anatomical variants, producing 120 independent model assessments. Architectures such as SqueezeNet1.0 and VGG16 consistently showed the weakest performance across evaluation settings (Appendix D).

**Fig. 2:**
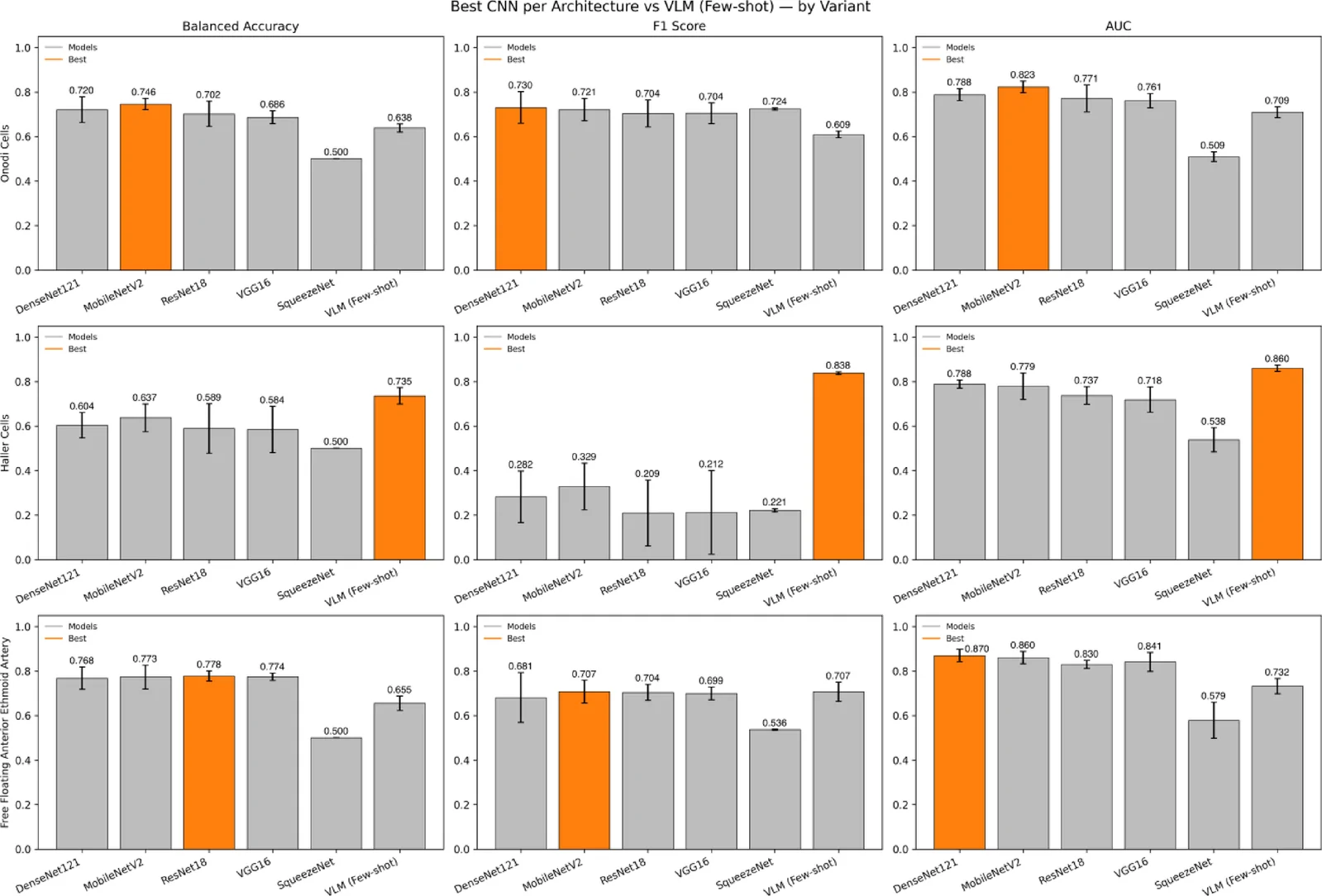
Comparative performance of CNNs and a VLM on three endoscopic-surgery - relevant CT classification tasks. Rows correspond to anatomical variants - Onodi Cells, Haller Cells, and FFAEA - and columns to evaluation metrics: Balanced Accuracy, F1 score, and AUC (all reported as decimals, 0–1). For each variant, bars show the best configuration within each CNN architecture family (DenseNet121, MobileNetV2, ResNet18, VGG16, SqueezeNet), selected by the highest mean balanced accuracy across all hyperparameter variants in the cross-validated runs, alongside the VLM (few-shot) model (BioMedCLIP). Orange bars highlight the best performer in each subplot; all other bars are gray. Error bars denote variability across internal repeated patient-wise 3 × 5-fold cross-validation. No independent external test set was used.

For each anatomical variant, the highest-performing configuration was selected to represent the benchmark CNN result. Among these top configurations, the most frequently occurring components were the MobileNetV2 architecture, the Adam optimiser, a LR of 0.001, and 15 training epochs. The final selected models and their corresponding performance metrics are summarised in Table 1; Fig. 2.

For FFAEA, the best-performing model was ResNet18 trained with Adam, a LR of 0.001, and 25 epochs, achieving a balanced accuracy of 77.8 ± 2.3%, an F1 score of 70.4 ± 3.6%, and an AUC of 83.0 ± 1.8%. For Haller cells, the top configuration employed MobileNetV2 trained with SGD, a LR of 0.0005, and 15 epochs, yielding a balanced accuracy of 63.7 ± 6.2%, an F1 score of 32.9 ± 10.5%, and an AUC of 77.9 ± 5.9%. For Onodi cells, the best-performing model used MobileNetV2 with Adam, a LR of 0.001, and 15 epochs, achieving a balanced accuracy of 74.6 ± 2.5%, an F1 score of 72.1 ± 5.1%, and an AUC of 82.3 ± 2.6%.

**Table 1:** Summary of best-performing CNN configurations for each anatomical variant under internal repeated patient-wise five-fold cross-validation.

Anatomical variant	Architecture	Optimizer	LR	Epochs	Balanced accuracy	F1 Score	AUC
FFAEA	ResNet18	Adam	0.001	25	0.778 ± 0.023	0.704 ± 0.036	0.830 ± 0.018
Haller Cell	MobileNetV2	SGD	0.0005	15	0.637 ± 0.062	0.329 ± 0.105	0.779 ± 0.059
Onodi Cell	MobileNetV2	Adam	0.001	15	0.746 ± 0.025	0.721 ± 0.051	0.823 ± 0.026

All performance metrics are reported as the mean of three five-fold cross-validation cycles for the specified configuration, with values presented to three decimal places as mean ± standard deviation. AUC = area under the receiver operating characteristic curve.

Following the 3 × 5-fold cross-validation evaluation, the BioMedCLIP-based classifier achieved its strongest performance in detecting Haller cells, with a balanced accuracy of 73.5 ± 3.6%, an F1 score of 83.8 ± 0.6%, and an AUC of 86.0 ± 1.4%. Performance for FFAEA was moderate, with a balanced accuracy of 65.5 ± 3.2%, an F1 score of 70.7 ± 4.3%, and an AUC of 73.2 ± 3.4%. The most challenging anatomical variant for the model was the Onodi cell, where balanced accuracy reached 63.8 ± 1.8%, accompanied by an F1 score of 60.9 ± 1.5% and an AUC of 70.9 ± 2.4% (Table 2, Fig. 2).

**Table 2:** Internal repeated patient-wise five-fold cross-validation performance of the BioMedCLIP few-shot image-embedding classifier for each anatomical variant.

Anatomical Variant	Setting	Balanced Accuracy	F1 Score	AUC
FFAEA	Few-Shot	0.655 ± 0.032	0.707 ± 0.043	0.732 ± 0.034
Haller Cell	Few-Shot	0.735 ± 0.036	0.838 ± 0.006	0.860 ± 0.014
Onodi Cell	Few-Shot	0.638 ± 0.018	0.609 ± 0.015	0.709 ± 0.024

All metrics are reported as the mean of three repeated five-fold cross-validation cycles, shown as mean ± standard deviation. AUC = area under the receiver operating characteristic curve.

## Discussion

This study explored the feasibility of using CNNs and VLMs to automate the identification of three anatomical variants with clinical relevance to ESS on CT sinus scans, including FFAEA, Onodi cells and Haller cells. We developed binary classification models for each variant using five CNN architectures with combinations of three different hyperparameters, including optimiser choice, learning rate and the number of training epochs, and we additionally assessed VLMs to determine whether multimodal embeddings improved discrimination. Five-fold cross-validation was employed to evaluate the models. Overall, the CNNs achieved moderate-to-good internal discrimination for FFAEA and Onodi cells, whereas CNN-based Haller cell detection proved more challenging. The BioMedCLIP few-shot image-embedding classifier showed complementary performance, particularly for Haller cells, but these results remain internally validated and require external confirmation.

### Interpretation of results

#### Patient and CT image distribution

The incidence of anatomical variants in our patient cohort differed slightly from the published ranges of FFAEA (20–42%), Onodi cells (8–33%), and Haller cells (10–30%) on CT sinus imaging^12–15,18^. In 122 patients referred for ESS, we identified FFAEA in 22%, Onodi cells in 24%, and Haller cells in only 7% of scans (Appendix C). Aside from random chance, this lower prevalence of Haller cells may also be attributed to the underlying disease profile of our cohort: many referrals were for CRS, a condition characterised by maxillary and ethmoidal mucosal thickening and opacification. Because scans with sinus opacification were included and no formal exclusion criterion was applied for anatomical obscuration, the lower observed prevalence of Haller cells may partly reflect reduced visibility of the infraorbital recess and maxillary infundibulum in patients with inflammatory mucosal disease. Such inflammatory change can obscure the infraorbital recess and infundibulum on CT, reducing the visibility of Haller cells and occasionally masking them completely^33^.

Notably, our study worked with the inherent assumption that the surgeons’ final categorisations served as the ground truth for the CNN. Future work would aim to recruit head-and-neck radiologists to oversee both CT image collection and scan validation, providing a more robust and standardised reference point. We would also aim to prospectively record each patient’s disease status and indication for ESS, allowing us to examine how often each variant is visible in different clinical contexts, and better understand the effects of underlying pathology on both radiological detection and CNN performance.

Across all three anatomical variants, more coronal images were extracted per patient when the variant was present compared with when it was absent. For FFAEA, an average of 5.00 coronal images were extracted per patient with the variant present, versus 2.91 when it was not present. For Haller cells, this was 5.88 vs. 3.19, and for Onodi cells, 5.03 vs. 3.85 (Appendix C). This approach was used to ensure that the full extent of each anatomical variant was captured and adequately represented, while fewer images were deemed sufficient to confirm typical anatomy in *Not Present* cases. Although images were grouped by patient ID during cross-validation to prevent patient overlap between training and validation sets, the CNN was trained on individual, independently labelled images, with no access to patient identifiers or the number of images per patient. Because all images from the same patient were kept within the same cross-validation fold, this design prevented patient-level leakage between training and validation sets. Nevertheless, the CNNs were trained on individual CT images, and differences in the number of images per patient and per class may have altered the effective image-level sample distribution. Future work should consider fixed image sampling per patient, patient-level aggregation, or volumetric DICOM-based modelling to reduce this potential source of bias.

#### Performance of all CNN configurations

When examining the performance metrics of all forty training configurations, a clear heterogeneity in results is evident (Appendix D). Despite this, two consistent patterns emerge: Poor F1 scores for Haller cell classification, and underperformance of the SqueezeNet1.0 and VGG16 architectures across all anatomical variants.

Haller cell classification consistently yielded the lowest performance, particularly in F1 score, despite seemingly acceptable AUC values (Appendix D). This discrepancy likely results from severe class imbalance: with only eight *Present* cases, the CNN encountered far more examples of the *Not Present* class during training. Consequently, the model became proficient at recognising the majority class (improving specificity and boosting AUC), yet struggled to detect rare *Present* cases, significantly lowering recall and thereby reducing the F1 score.

Understanding performance differences across architectures requires insight into the mechanisms of DL. CNNs extract features hierarchically: early layers detect edges and corners, intermediate layers capture shapes and contours, and deeper layers distinguish task-specific features like anatomical structures. Consequently, architecture depth - the number of layers - directly affects a CNN’s capacity to learn the spatial patterns needed for accurate classification.

SqueezeNet1.0, a notably shallow architecture, has limited depth and struggles to capture subtle image details. In contrast, VGG16 is substantially deeper and more prone to overfitting, especially when training data is limited. Both extremes can impair performance: shallow models underfit by failing to learn key patterns, while overly deep models overfit by memorising extraneous patterns. For example, Yang et al. found that SqueezeNet1.0 had the lowest accuracy in COVID-19 lung CT classification due to poor feature extraction, while VGG16 showed poor generalisation, likely from overfitting to patient-specific features^34^. In addition, using adaptive optimisers such as Adam can exacerbate overfitting in deeper architectures like VGG16, leading to poorer performance; this phenomenon was illustrated in our study (Appendix D).

Intermediate-depth CNN architectures - including MobileNetV2, ResNet18, and DenseNet121 - strike a favourable balance, offering enough layers to learn meaningful anatomical patterns without significantly overfitting. This likely explains why MobileNetV2 and ResNet18 featured most frequently among the top-performing training configurations in our classification tasks.

#### Summary of best-performing CNN configurations

Among the best-performing training configurations, clear patterns were observed (Table 1). MobileNetV2 and ResNet18 were the best-performing architectures, consistent with earlier observations that networks of moderate depth can learn subtle features without overfitting. Notably, both architectures are computationally lightweight, which may facilitate future evaluation in resource-constrained environments. However, given the current 2D coronal CT-image workflow, absence of recorded DICOM geometry, non-standardised PACS windowing, and lack of external validation, the present models should not yet be considered deployment-ready.

Adam was the better optimiser for FFAEA and Onodi cell classification, with its adaptive nature helping the model pick up useful patterns quickly. In contrast, SGD was superior for Haller cell classification, where the small *Present* class benefited from the optimiser’s steadier updates. A LR of 0.001 worked best for FFAEA and Onodi cells, while the lower rate of 0.0005 was preferable for Haller cells, reducing the risk of overshooting useful features in a scarce *Present* dataset. An epoch count of 15 was sufficient for Haller and Onodi cells, preventing over-training, whereas 25 epochs improved FFAEA performance, which - being the least imbalanced variant in our dataset (Table 1) - could withstand more iterations without overfitting.

Interpreting our metrics against published benchmarks suggests that the internal performance of the best CNNs for FFAEA and Onodi cells falls within ranges reported for some prior binary classification models in medical imaging^35–39^. However, direct comparison across studies is limited by differences in imaging modality, task difficulty, dataset size, validation design, and class balance. Accordingly, these results support feasibility rather than clinical utility^32,40^. External validation on native DICOM data and comparison against specialist radiological assessment will be required before clinical applicability can be established.

#### Comparative performance of CNNs and the VLMs

A complementary analysis with a few-shot VLM provides additional insight into how large multimodal priors interact with these tasks. For Haller cells, the few-shot VLM achieved a balanced accuracy of approximately 0.735, an AUC of 0.860, and an F1 score of 0.838, consistently exceeding the performance of our best CNN configurations selected within each architecture family by balanced accuracy. This pattern suggests that the VLM’s semantic priors align closely with the morphological cues of Haller cells, enabling effective generalisation under limited task-specific supervision. In contrast, for Onodi cells and FFAEA, the best CNNs retained an advantage on threshold-free and class-balance-aware measures, with top runs commonly attaining AUC values around 0.80–0.87 and balanced accuracy above 0.73, whereas the VLM achieved an AUC of 0.709 and balanced accuracy of 0.638 for Onodi cells, and an AUC of 0.732 and balanced accuracy of 0.655 for FFAEA. Notably, the VLM’s F1 scores remained competitive or higher in these latter tasks (approximately 0.609 for Onodi cells and 0.707 for FFAEA), indicating a tendency toward recall-heavy operating points that favour detection of positives at the expense of specificity.

These disease-specific outcomes are consistent with the differing inductive biases of the two paradigms. VLMs bring language-aligned priors derived from large, heterogeneous pretraining corpora that can act as strong regularisers when disease morphology is semantically captured by the pretraining distribution, as appears to be the case for Haller cells. When the discriminative cues are more subtle, spatially small, or domain-specific - features characteristic of Onodi cells and the free-floating anterior ethmoid artery - task-optimised CNNs trained end-to-end on the target distribution tend to produce superior ROC characteristics and class-balanced performance. The divergence between higher VLM F1 and lower balanced accuracy/AUC in some settings also points to calibration differences: a model can realise a high F1 by operating at a threshold that strongly favours recall on an imbalanced dataset, while still yielding suboptimal discrimination when assessed across thresholds or balanced between classes. Because BioMedCLIP was used through its pretrained image encoder without text prompts or language-encoder features, these experiments should be interpreted as few-shot classification using biomedical image embeddings rather than as a full multimodal reasoning workflow.

From a translational perspective, these complementary error profiles identify promising directions for future research rather than immediate deployment strategies. The BioMedCLIP image-embedding approach may warrant further evaluation as an adjunctive detector for Haller cells, whereas CNNs presently appear more reliable for FFAEA and Onodi cells under internal validation. However, because the present study used 2D coronal CT inputs from a single centre, did not record DICOM geometry or enforce standardised windowing, and lacked external validation, neither approach should yet be considered ready for clinical implementation.

### Limitations and future directions

#### Dataset and class imbalance

Our dataset of 122 single-centre CT sinus scans is modest compared to other CNN studies assessing the same imaging modality, which have analysed between 240 and 1300 scans^32,41^. Our smaller sample size limits external validity and increases the risk of overfitting, as the CNN is exposed to too few *Present* examples to generalise meaningful patterns, tending towards memorising them instead. It also meant that we were unable to allocate scans for an independent test set and instead relied solely on training and validation folds. Class imbalance was also a challenge, with *Not Present* CT images being 2.0-7.7 times more frequent than *Present* across the three anatomical variants (Appendix C). If left unaddressed, the CNN can default to predicting the majority class (*Not Present*), resulting in misleadingly high accuracy but poor detection of the minority class (*Present*). These dataset constraints similarly affected the VLM experiments, as VLMs tend to require larger and more heterogeneous datasets to develop stable radiological representations.

Importantly, all reported performance metrics are internal single-centre estimates obtained through repeated patient-wise cross-validation rather than evaluation on an independent hold-out or external cohort. Although patient-wise cross-validation reduces the risk of data leakage relative to image-level splitting and is appropriate for small datasets, it does not substitute for external validation. Accordingly, the generalisability of both the CNNs and the BioMedCLIP image-embedding classifier across institutions, scanners, acquisition protocols, reconstruction settings, PACS environments, and patient populations remains unproven.

We adopted multiple strategies to combat these risks. Five-fold cross-validation was used to obtain more robust performance estimates; by iteratively training on 80% and validating on 20% of the data in five rotations, we ensured the CNN was exposed to the entire dataset for training. This approach, also widely used in other medical AI studies, improves generalisation to unseen data and reduces the risk of overfitting, especially for small datasets^42–44^. Data augmentation was applied to expand the dataset, a strategy known to accelerate training and reduce overfitting by increasing image variability^36^. Weighted sampling during batch selection helped address class imbalance, as shown by Kumar et al., who reported improved CNN performance on imbalanced medical datasets^45^. Finally, evaluating balanced accuracy rather than standard accuracy ensured model performance was fairly represented across both classes, preventing artificially inflated results due to the over-representation of the *Not Present* class.

While these strategies help mitigate the effects of class imbalance and limited data, they do not eliminate them entirely. A more robust solution would involve building a substantially larger dataset - with at least a hundred or more images in both the *Present* and *Not Present* classes for each anatomical variant - as seen in previously published binary classification studies^26,32,45^. Such an expansion would also allow more meaningful benchmarking of VLMs, which generally rely on broader datasets to overcome their weaker performance on subtle grayscale anatomical features. This would also support the use of an independent test set and open the door to further validation, including comparisons of CNN performance against head-and-neck radiologists.

#### Binary classification and 2D imaging

As described in the Methods, this proof-of-concept study used 2D coronal CT images curated from PACS rather than native DICOM volumes. Raw DICOM metadata were not extracted, and the coronal reformat matrix, pixel spacing, slice thickness, and slice spacing were not recorded for individual scans. Although no spatial resampling was performed before image capture, images were acquired at the native PACS viewing resolution using the default bone or sinus window settings available at the time of review, and a fixed window width/level preset was not enforced. Consequently, the models did not access original Hounsfield unit information, acquisition metadata, or standardised voxel geometry. These factors may affect reproducibility and transferability, particularly for small structures such as the anterior ethmoidal artery.

In addition, a fixed interval between consecutive CT images was not enforced. Instead, the most anterior slice in each stack was standardised using anatomical landmarks, and additional posterior images were selected by clinical judgement to represent the relevant anatomy. This multi-image approach preserved some spatial context and extended previous single-image approaches, but it could not replicate volumetric assessment and may have introduced observer-dependent image-selection variability.

Although the selected variants can be visualised on coronal CT, routine ESS planning commonly confirms Onodi cells and the exact course of the anterior ethmoidal artery using axial and sagittal reformats. The absence of multi-planar and volumetric context in the present study likely reduced annotation certainty and model ceiling performance, particularly for subtle or spatially complex variants.

Moreover, several other variants relevant to ESS planning require assessment beyond the coronal plane. For example, surgeons often refer to the CLOSE acronym for pre-operative ESS planning, a mnemonic that flags five high-risk structures on sinus CT (Cribriform plate, Lamina papyracea, Onodi cell, Sphenoid sinus, and Ethmoid artery). The final letter - *S* for sphenoid sinus pneumatisation - refers to three patterns (conchal, presellar, and sellar), which can only be assessed in the sagittal plane^9^. These limitations also affected VLMs, as their pretrained embeddings lack explicit 3D anatomical awareness and are not optimised for subtle radiological density variations across planes.

This three-group categorisation highlights another limitation of our study: the CNN relied on binary classification, labelling each variant as simply present or absent. In reality, these structures can be grouped into multiple categories, or vary along a spectrum of size and position, each carrying a different operative risk. For example, the FFAEA may adhere closely to the skull base (grade I), protrude slightly (grade II), or hang well below the ethmoid roof (grade III), with deeper descent markedly increasing the chance of arterial injury^11,46^. This issue is particularly relevant for FFAEA, where there is no universally accepted millimetric definition of *free-floating*; our binary labels therefore reflected expert visual consensus rather than a graded anatomical severity scale. A similar three-tier system, known as the Keros classification, grades olfactory fossa depth and forms part of the CLOSE checklist; a deeper fossa (Keros III) lowers the cribriform plate and increases the risk of CSF leak^9^. Likewise, Haller cells range from small infra-orbital air cells of little consequence to large pneumatisations that narrow the maxillary infundibulum, and Onodi cells can barely protrude into - or markedly encroach on - the sphenoid sinus, each scenario influencing operative exposure and complication risk^47^. Although our model may assist in flagging high-risk variants for radiologist review, it lacks the ability to capture finer anatomical nuances, leaving much of the pre-operative assessment reliant on manual interpretation. VLMs also struggle with this nuance, as they are not trained to recognise graded anatomical variants and do not inherently model the clinical significance associated with different morphological subtypes.

Future work should build on our model by incorporating 3D imaging data and applying segmentation: an approach that enables precise localisation and measurement of anatomical structures on scans. This has shown successful implementation in previous radiological studies, including those focused on CT sinus imaging^41^. In the absence of 3D data, a promising alternative would be to explore 2.5D inputs. This technique, described in a 2022 study that developed a CNN to distinguish between HPV-positive and negative oropharyngeal cancers on CT, involved stacking axial, sagittal, and coronal slices taken at the same anatomical landmark as three input channels. This provided greater spatial context for the model and yielded strong performance metrics, demonstrating the value of the approach. Future research could also investigate radiology-specific fine-tuning of VLMs or hybrid CNN-VLM pipelines, which may improve anatomical understanding beyond what general-purpose VLMs currently achieve.

## Conclusions

This study demonstrates the feasibility of using CNNs and a pretrained biomedical image-embedding model to identify selected ESS-relevant anatomical variants on standardised coronal CT images. Across repeated patient-wise cross-validation, CNN-based models achieved the strongest performance for detecting free-floating anterior ethmoidal arteries and Onodi cells, with balanced accuracies of 77.8% and 74.6%, respectively, and AUC values above 82.0%. In contrast, the BioMedCLIP few-shot image-embedding classifier showed the best performance for Haller cell detection, achieving a balanced accuracy of 73.5% and an AUC of 86.0%. These findings suggest that task-optimised CNNs and biomedical foundation-model embeddings may offer complementary strengths for automated assessment of surgically relevant sinonasal anatomy. By benchmarking multiple CNN architectures alongside a pretrained biomedical vision-language model, this work provides an initial framework for AI-assisted identification of anatomical variants that are frequently underreported but important for operative planning. The results support the potential role of automated image analysis as an adjunct to radiological and surgical review, particularly for flagging variants associated with increased risk during endoscopic sinus surgery. Future development should focus on DICOM-native, multi-planar or volumetric modelling, larger multi-centre datasets, and independent external validation to support translation toward clinically robust pre-operative decision-support tools.

## Supplementary Information

Below is the link to the electronic supplementary material.

Supplementary Material 1

Supplementary Material 2

Supplementary Material 3

Supplementary Material 4

## Data Availability

All anonymised CT image data, model training scripts, and final pretrained weights for each of the 40 CNN configurations - including code for image preprocessing, augmentation, cross‐validation splits, and performance evaluation - have been deposited in a public Bitbucket repository at: https://bitbucket.org/iAnalytica/ai-driven-nose-ct-classification. This repository also contains detailed README instructions to reproduce our training and validation workflows.
