# Supplementary material for: Deep learning classification of nasal anatomical variants on computed tomography for endoscopic sinus surgery
Source: Sci Rep. 2026 Jul 1;16:30235. doi: 10.1038/s41598-026-59617-1 (PMC13623651; doi:10.1038/s41598-026-59617-1)
Supplement: Supplementary file 2 [file 41598_2026_59617_MOESM1_ESM.docx]

Appendix C

Table 1 and 2 summarize the occurrence of three key sinonasal anatomical variants - FFAEA, Haller cell, and Onodi cell - both across the full patient cohort (n = 122) and within individual CT image stacks. At the patient level, FFAEA was present in 27/122 (22.1%), Haller cells in 8/122 (6.6%), and Onodi cells in 29/122 (23.8%). When evaluated on a per‐slice basis, the anterior stack (n = 411) contained FFAEA in 135 slices (32.8%) and Haller cells in 47 slices (11.4%), while in the posterior stack (n = 504), Onodi cells appeared in 146 slices (29.0%).

**Table 1.** Prevalence of Anatomical Variants at the Patient Level.

| **Patients (n=122)** | **Present** | **Not Present** | **Total** |
| --- | --- | --- | --- |
| FFAEA | 27 | 95 | 122 |
| Haller Cell | 8 | 114 | 122 |
| Onodi Cell | 29 | 93 | 122 |
| **Total** | 64 | 302 | 366 |

**Table 2.** Prevalence of Anatomical Variants at the Imagery Level.

| **CT Images (n=915)** | **Present** | **Not Present** | **Total** |
| --- | --- | --- | --- |
| FFAEA (Anterior Stack) | 135 | 276 | 411 |
| Haller Cell (Anterior Stack) | 47 | 364 | 411 |
| Onodi Cell (Posterior Stack) | 146 | 358 | 504 |
| **Total** | 328 | 998 | 1,326 |
