# Supplementary material for: Deep learning classification of nasal anatomical variants on computed tomography for endoscopic sinus surgery
Source: Sci Rep. 2026 Jul 1;16:30235. doi: 10.1038/s41598-026-59617-1 (PMC13623651; doi:10.1038/s41598-026-59617-1)
Supplement: Supplementary file 3 [file 41598_2026_59617_MOESM3_ESM.docx]

**Appendix B**

To rigorously assess model generalizability and prevent patient-level information leakage, scans from the same patient were always assigned to the same fold; the cohort was first stratified by class label and then randomly shuffled (with a unique seed per cycle) into five disjoint folds (F1-F5), with four folds (80% of patients) used for training and the remaining fold (20%) for validation in each split (S1-S5). This process was repeated such that each fold served once as the hold-out set, yielding five performance estimates per cycle, and the entire five-fold procedure was further repeated across three independent cycles to average out variance due to random partitioning. Performance metrics (accuracy, sensitivity, specificity, area under the ROC curve) were computed for each hold-out split and averaged across all folds and cycles to provide the final mean ± standard deviation estimate of expected generalization. Figure 1 illustrates this protocol: each box represents a fold containing patient scans, *Random Shuffle* indicates the seed-based allocation of scans into folds.

**Figure 1.** Illustration of the patient-wise repeated stratified five-fold cross-validation method. Each box represents a fold containing patient scans. Random Shuffle refers to the random allocation of scans into folds based on the seed value used for that cycle. F = fold; S = split.
