# Supplementary material for: Deep learning classification of nasal anatomical variants on computed tomography for endoscopic sinus surgery
Source: Sci Rep. 2026 Jul 1;16:30235. doi: 10.1038/s41598-026-59617-1 (PMC13623651; doi:10.1038/s41598-026-59617-1)
Supplement: Supplementary file 4 [file 41598_2026_59617_MOESM4_ESM.docx]

Appendix D

To systematically compare the detection performance of five state-of-the-art CNN backbones on sinonasal anatomical variants, we computed AUC, F1 score, and balanced accuracy for each model on the held-out folds of our patient-wise repeated stratified five-fold cross-validation (see Appendix B). The resulting heatmap (Figure 1) displays the mean metric values over all folds and random-seed cycles.

**Figure 1.** Heatmap demonstrating performance metrics for all training configurations. Left: The 40 training configurations, with modifiers listed in the order of architecture, optimiser, learning rate and epoch count. Divisions are made between each architecture. Top: Anatomical variants. Bottom: Performance metrics. Right: Colour bar keyed to the mean value of each metric. All performance metrics are reported as the mean of three five-fold cross-validation cycles for the specified configuration, with values presented to three decimal places as mean ± standard deviation.

SGD = Stochastic gradient descent; LR = learning rate; ep = number of training epochs; AUC = Area under the receiver operating characteristic curve.
